# Acetylcholinesterases from Leaf-Cutting ant* Atta sexdens*: Purification, Characterization, and Capillary Reactors for On-Flow Assays

**DOI:** 10.1155/2019/6139863

**Published:** 2019-07-01

**Authors:** Adriana M. Dos Santos, Ariele C. Moreira, Bianca Rebelo Lopes, Mariana F. Fracola, Fernando G. de Almeida, Odair C. Bueno, Quezia B. Cass, Dulce Helena F. Souza

**Affiliations:** ^1^Federal University of São Carlos, Department of Chemistry, São Carlos, SP, Brazil; ^2^São Paulo University, Instituto de Ciências Biomédicas (ICB), São Paulo, Brazil; ^3^São Paulo State University, Center for the Study of Social Insects, Rio Claro, SP, Brazil

## Abstract

Acetylcholinesterase (AChE) is responsible for catalyzing the hydrolysis of the neurotransmitter acetylcholine (ACh) leading to acetate and choline (Ch) release. The inhibition of AChE produces a generalized synaptic collapse that can lead to insect death. Herein we report for the first time the isolation of two AChEs from* Atta sexdens* which were purified by sulphate ammonium precipitation followed by ion exchange chromatography. AsAChE-A and AsAChE-B enzymes have optimum pH of 9.5 and 9.0 and higher activities in 30/50°C and 20°C, respectively, using acetylthiocholine (ATCh) as substrate. Immobilized capillary enzyme reactors (ICERs) were obtained for both enzymes (AsAChE-A-ICER and AsAChE-B-ICER) and their activities were measured by LC-MS/MS through hydrolysis product quantification of the natural substrate ACh. The comparison of activities by LC-MS/MS of both AChEs using ACh as substrate showed that AsAChE-B (free or immobilized) had the highest affinity. The inverse result was observed when the colorimetric assay (Elman method) was used for ATCh as substrate. Moreover, by mass spectrometry and phylogenetic studies, AsAChE-A and AsAChE-B were classified as belonging to AChE-2 and AChE-1 classes, respectively.

## 1. Introduction

The ecological importance of ants is indisputable due to the effect/influence they have on several processes such as aeration, distribution of nutrients, and seed dispersal [1, 2]. The leaf-cutting ants harvest fresh leaves to cultivate the symbiotic fungi that serve as the base for feeding the colony [3–5]. Controversially, some species of ants increase their population density especially in environments where there is a reduction in biodiversity, such as those designated for agriculture, forestry development, or construction of cities [1]. The genus* Atta *(Hymenoptera: Formicidae) stands out for economic importance because of their destructive power of a large number of plant species [6, 7] causing direct and indirect economic damage to agriculture [8, 9], pasture, and silviculture [10]. The most efficient method to control leaf-cutting ants is by chemical control using chemical components that, in most case, are not selective and can harm human health and the environment [11].

Acetylcholinesterase (AChE) (EC 3.1.1.7) is a cholinesterase that acts on the central nervous system and plays an important role during neurotransmission in the cholinergic synapses and neuromuscular junctions. It is being responsible for the hydrolysis of the active neurotransmitter acetylcholine (ACh) into the inactive compounds choline (Ch) and acetic acid. These enzymes are secreted as soluble form or membrane-anchored by a hydrophobic domain [12].

Concerning insects in general, two AChEs coming from distinct genes (locus ace 1 and locus ace 2) have been described. The two different* ace *loci generate distinct enzymes; however, AChE can assume multiple molecular forms differentiated by the number and types of subunits providing a functional diversification of the enzyme [13–15]. Locus ace 1 codes for AChE1, which is the main synaptic enzyme involved in the transmission of the cholinergic signal. Ace 2 locus codes for AChE2 that has limited cholinergic function, exhibits other noncholinergic functions [16–20], and may be directly related to the resistance to insecticides [21–23]. Insect AChE mode of action is not well established and reports have shown that in some insects, such as* Bombyx mori* and* Apis mellifera,* AChE2 is the main catalytic enzyme in synaptic transmission rather than AChE1 [14, 24], while* Drosophila melanogaster* and* Musca domestica* only have the ace 2 gene [25].

AChE inhibition can lead to a generalized synaptic collapse causing the insect to die; thus this enzyme has been exploited as a molecular target for the development of insecticides [26, 27]. The most usual classes of compounds are organophosphates and carbamates, both of which act by inactivation of the AChE serine residue (residue present in the active site and important for catalysis). It is worth mentioning that organophosphate and carbamate insecticides are highly toxic to animals and humans [28–31].

To contribute to this field, this work herein reports on the isolation and characterization of two AChEs from* Atta sexdens. *Moreover, the purified enzymes were used to produce immobilized capillary enzyme reactors (ICERs) to prospect inhibitors based on the direct hydrolysis of ACh and quantification of the produced choline by LC-MS/MS.

## 2. Material and Methods

### 2.1. Biological Samples

*A. sexdens *Linneaus (Hymenoptera: Formicidae) was collected from a laboratory nest located at the Social Insects Study Center (UNESP, Rio Claro, Brazil). It was supplied daily with* Eucalyptus alba* leaves, oat seeds, and occasionally leaves from other plants such as* Hibiscus* sp.,* Ligustrum* sp., or rosebush petals. After collection, the ants were stored at -80°C until use. Heads from worker (1 g) were macerated in 10 mL of 50 mM phosphate buffer pH 8.0 (buffer A), centrifuged for 5 min at 1,500 g to provide the supernatant that was called crude extract.

### 2.2. Enzymes Purification

#### 2.2.1. Ammonium Sulfate Precipitation

The crude extract was precipitated with 55% (w/v) ammonium sulfate at room temperature and was continuously stirred for 5 min. Thereafter, the suspension was kept under static condition for 1 h and centrifuged at 10,300 g for 1 h. The pellet was resuspended in 2.5 mL of buffer A following dialysis on a Minidialysis device 3.5k MWCO (Thermo Scientific) for 16 h at 4°C against buffer A, which was changed three times.

#### 2.2.2. Ion Exchange Chromatography

The dialyzed sample (2 mL) was purified by anion exchange chromatography on a HiTrap DEAE-FF column (1 mL) previously equilibrated with buffer A in an AKTA-FPLC™ system (GE Healthcare Sciences). The elution was made with nonlinear gradient with buffer B (buffer A plus 1 M NaCl) consisting of the 5 steps at 10, 20, 40, 60, and 100% buffer B. Each gradient ramp was made with 2 mL of buffer maintaining 10 mL of buffer between the steps. The separation was carried out at 0.6 mL.min^−1^ and 1 mL fractions were collected. Fractions from the same peak with AChE activity measured by Ellman's protocol [32] were pooled, concentrated, and dialyzed against buffer A using an Amicon® Ultra-15-10,000NMWL (Millipore) to the final volume of 1 mL. The samples were used for enzymatic characterization as free enzyme and for producing the ICERs.

### 2.3. Enzyme Concentration

The protein concentration was determined by the Bradford assay [33] using the Bio-Rad protein assay kit containing Coomassie Brillant Blue G250 (Bio-Rad Laboratories) and bovine serum albumin (BSA) as the standard.

### 2.4. Gel Electrophoresis and Zymography Analysis

Enzyme purification was followed by electrophoresis in 15% (w/v) native-PAGE (Laemmli, 1970). The native–PAGE was accomplished in the absence of denaturing agents (2-mercaptoethanol; and sodium dodecyl sulfate) and the samples were not heated prior the run. After the run, the gel was stained with Coomassie Blue.

In–gel zymography was used to determine the AChE activity, using a 15% native-PAGE. After gel running, the gel was equilibrated with 0.5 M Tris-HCl buffer pH 8.0 at room temperature during 45 min with buffer changes every 15 min. The gel was incubated for 30 min with 0.5 M Tris-HCl buffer pH 8.0 containing 0.3 mM acetylthiocholine iodide (ATChI) and 0.3 mM 5,5′-dithiobis(2-nitrobenzoic acid) (DTNB).

### 2.5. Identification of Isolated Enzymes by Mass Spectrometry Analysis

The isolated enzymes were identified by mass spectrometry from gel-tryptic digestion. To do this, sample bands were excised from Coomassie stained native-PAGE and were tryptic cleaved [34]. ZipTips® were used for desalting and samples kept at −20°C. The LC–MS/MS analysis was performed as previously described [35, 36]. Databases with different numbers of sequences were used to increase the protein identification confidence.

AChE1 and AChE2 sequences from insects were retrieved from the GenBank at the National Center for Biotechnology Information (NCBI) website (https://www.ncbi.nlm.nih.gov/genbank/). The sequence alignments against other insect AChEs were carried out using the Clustal Omega (https://www.ebi.ac.uk/Tools/msa/clustalo/). Phylogenetic trees were constructed by the* Phylogeny.fr* [37] and* MEGA7* [38].

### 2.6. Characterization and Kinetic Studies of Free Enzymes

#### 2.6.1. Enzymatic Assays

The cholinesterase activity was evaluated by the Ellman method [32] using acetylthiocholine (ATCh) as substrate. The enzymatic reaction consisted of 30 *μ*l of crude extract or 50 *μ*l of purified fractions in 750 *μ*L of reaction mixture (50 mM Tris-HCl buffer pH 8.0, 0.3 mM ATChI and 0.3 mM DTNB) and the absorbance was monitored at 412 nm. One unit of AChE activity was defined as the amount of enzyme that hydrolyzes 1 *μ*M of substrate per minute. Each sample was analyzed in triplicate.

#### 2.6.2. Influence of the Temperature and pH on the Enzymatic Activity

To evaluate the influence of temperature on enzyme activity, the assays were performed at different temperatures from 10°C to 60°C. The effect of pH on the activity of AChE was evaluated using three different buffers, with two points intersecting two different buffers (McIlvaine buffer pH 5.0-6.5, 50 mM; sodium phosphate buffer pH 6.5-8.0 and 50 mM Tris–HCl buffer pH 8.0-9.5). Activities were plotted against temperature or pH values, respectively. Each sample was analyzed in triplicate.

#### 2.6.3. Kinetic Studies

The kinetic parameters were evaluated in the optimal conditions of pH and the temperature previously determined. The AsAChE-A (0.84 U) and AsAChE-B (0.17 U) activities were evaluated by varying the substrate ATCh concentrations (10 to 250 *μ*M). The experiments were carried out in triplicate. Michaelis-Menten constants (K_M_) and maximum velocities (V_max_) were estimated through Lineweaver-Burk reciprocal plots using GraphPad Prism 5.0 software.

### 2.7. Characterization and Kinetic Studies of Immobilized Enzymes

#### 2.7.1. Preparation of AsAChEs Immobilized Capillary Enzyme Reactors (ICERs)

AsAChEs eluted from the DEAE column were immobilized onto the internal surface of an open tubular silica capillary (100 *μ*m I.D. x 0.375 mm x 40 cm) as previously described by Vanzolini et al. [39] for AChE from* Electrophorus electricus *(*eel*AChE-ICER). The immobilization was carried out in duplicate to ensure the reproducibility of the produced ICERs.

#### 2.7.2. LC-MS System

The analyses were carried out using a LC system ACQUITY UPLC (Waters, Milford, USA) containing a binary pump (BSM) and a quaternary H-class pump (QSM), an automated injector Waters 2777C. The LC system was coupled to a Xevo TQ-MS (Waters, Milford, USA) mass spectrometer equipped with an ESI source operating in a positive ionization mode. MassLynx 4.1 software (Waters, Milford, USA) was used for data acquisition and processing.

The mass spectrometer was operated by selected reaction monitoring (SRM) in which the protonated molecular ion was isolate and the fragments ions were monitored to the choline and acetylcholine. Nitrogen was used as desolvation gas at 600 L/h at a temperature of 350°C. The capillary voltage was set at 2.0 kV and the collision gas flow at 0.15 mL/min. The activity and kinetic parameters were evaluated by one SRM transition for each analyte at the following cone voltage (CV) and collision energy (CE): 146.10 > 87.02 (CV = 10 V; CE = 12 eV) for ACh and 104.07 > 60.10 (CV = 10 V; CE = 20 eV) for Ch.

(*1) Chromatographic Conditions*. The* As*AChE-ICERs were used as the bioaffinity column with ammonium acetate solution (15 mM, pH 8.0) as the mobile phase at a flow rate of 0.05 mL.min^−1^ and injection sample volume of 10 *μ*L. Methanol was used in the combined mode to improve ionization which was delivered by the syringe pump at a flow rate of 0.05 mL. min^−1^. The total analysis time was of 8.0 min. All LC analyses were performed at room temperature (± 20°C).

To evaluate the stability of the ACh solution to spontaneous hydrolysis in the sample injector and in the pretreated capillary, a chromatographic separation under HILIC conditions was used. To meet this end, 10 *μ*L of ACh (60 *μ*M, ammonium acetate 15 mM; pH 5.0) was consecutively injected, every 10 min throughout a total period of 360 min, onto a CORTECS™ UPLC HILIC 2.7 *μ*m (2.1 x 100 mm) column with ACN:ammonium acetate (15 mM; pH 5.0.) (30:70 v/v) as the mobile phase at 0.5 mL.min^−1^ flow rate. Ch and ACh were monitored by SRM as described for the enzymatic hydrolysis with the ICERs. No Ch production was observed and the ACh maintained the same peak area with a carryover effect of 0.1% (n = 5).

#### 2.7.3. Analytical Method Qualification

The method qualification was asserted using calibration curves with two concentration ranges, in accordance with internationally accepted criteria (https://www.fda.gov/media/70858/download) (Supporting Information – A)

#### 2.7.4. Kinetics Studies of the AsAChE-ICERs

The ICERs kinetic parameters were determined by monitoring the production of Ch from duplicate injections, in the LC-MS/MS system, of 10 *μ*L of ACh (10.0 to 200.0 *μ*M) solutions.

The areas of the Ch peak produced were correlated to the concentrations through the calibration curves. The values obtained for Ch concentrations were related to substrate concentrations and the best-fit nonlinear regression method using the GraphPad Prism 5.0 software was used to obtain the Michaelis-Menten curve, thus determining the values of K_M_ and V_max_.

### 2.8. Enzymatic Assay of Free Enzymes with ACh as Substrate and Analysis by LC-MS/MS

The hydrolysis of the natural substrate ACh with the free enzyme was monitored in duplicate by LC-MS/MS. To do that, 10 *μ*L of the enzymatic solutions (AsAChE-A and AsAChE-B) previously dialyzed into the ammonium acetate buffer (15 mM, pH 5.0) were used and the reactions were carried out with 70 *μ*L of ammonium acetate buffer (15 mM, pH 5.0) and 20 *μ*L of the ACh solutions at the following concentrations: 22.8 *μ*M; 34.2 *μ*M; 45.6 *μ*M; 68.4 *μ*M; 114 *μ*M; 159.6 *μ*M; 205.2 *μ*M; and 239.4 *μ*M. The enzymatic reactions were stopped after 5 min by adding 100 *μ*L of acetonitrile (ACN) followed by centrifugation at 20,000 g for 5 minutes at room temperature. The supernatants were transferred to vials and injected with an injection volume of 10 *μ*L onto a CORTECS™ UPLC HILIC 2.7 *μ*m (2.1 x 100 mm) column with ACN:ammonium acetate (15 mM; pH 5.0.) (30:70 v/v) as the mobile phase at 0.5 mL.min^−1^ flow rate. ACh and Ch were monitored by SRM as described in Item 2.7.2 and the peak area for Ch was correlated to its concentrations through the calibration curves.

### 2.9. Screening Assays for Tacrine

Tacrine was used as the standard AChEIs for both AsAChR-ICERs. The substrate concentration used was 1.5 times the Km value, to ensure the saturation of the ICER.

For each AChEIs assay, 0.1 mM of tacrine was prepared in a total volume of 100 *μ*L with ammonium acetate buffer (15.0 mM, pH 8.0) containing 25 *μ*L of the ACh at 100 *μ*M, 283 *μ*M for ICER_AsAChE-A, and 200 *μ*M for ICER_AsAChE-B. For each analyzed sample, a negative control (absence of ACh) and positive control samples (absence of tacrine) were used. The inhibition percent I (%) for tacrine was calculated according to

(1) $$\begin{array}{c} I\left(\;\%\;\right)=100-\left(\;\frac{Pi}{P0}*100\;\right) \end{array}$$

where Pi is the Ch production quantified from the hydrolysis of acetylcholine in the presence of the tacrine and P0 is the Ch production of the positive control sample.

## 3. Results and Discussion

### 3.1. Enzymes Purification

Two AsChEs were purified from worker heads of* A. sexdens*. Medium worker ants were selected considering our previous studies, in which we identified higher AChE expression levels in this developmental stage, compared to larva and pupa [40].

No surfactant was used to extract both AChEs thus showing their hydrophilic characteristics. Similar results were reported for isolation from other insects of the Hymenoptera order such as* Apis mellifera* [14] and Nematoda* Heterorhabditis bacteriophora* [41].

The purification steps were accompanied by enzymatic activity of free enzyme with ATCh as substrate using Ellman's protocol [32]. To meet this end, the proteins were precipitated with ammonium sulfate.  Table 1 lists the activity recoveries for the purification protocol. A decrease in total protein concentration was obtained but with a recovery of 180% after pellet resuspension with a 4.0-fold purification increase. A recovery over 100% after precipitation can be associated with the elimination of inhibitors and intramolecular enzyme interactions [42].

**Figure 1:**
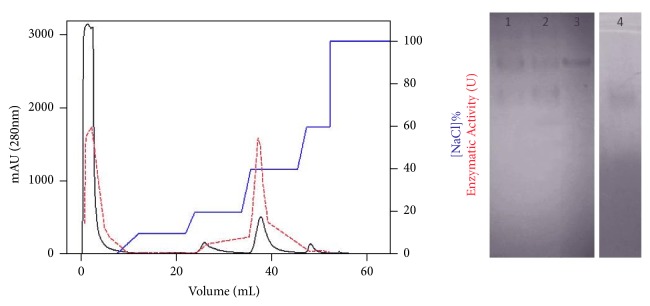
Purification and activity of AChEs from* A. sexdens *(AsAChE). Elution profile of ion exchange chromatography (left). Fractions were eluted with a nonlinear gradient of 50 mM phosphate buffer, 1 M NaCl, pH 8.0. AChE activity of free enzyme is shown with dotted line. Zymography on 15% native-PAGE using acetylthiocholine iodide (ATCh) as a substrate (right). Lane 1, crude extract; lane 2, fraction after sulphate ammonium precipitation and applied onto DEAE FF column; lane 3, AsAChE-B and lane 4, AsAChE-A.

The separation of two AChE active fractions was obtained by ion exchange chromatography. The first isolated fraction was excluded in the dead volume of the anionic column (AsAChE-A), while the second fraction was eluted only with about 40% of buffer B (AsAChE-B) (Figure 1). Moreover, the presence of these two enzymes was also identified by zymography of the crude ant head extracts, corresponding to the isolated AChEs (Figure 1).

### 3.2. Identification of the Isolated Enzymes by Mass Spectrometry

The list of identified peptides is summarized in Table 2. These data showed that both isolated enzymes were identified as AchEs. The identified peptides based on the phylogeny studies were also used to classify the enzymes in accordance with their classes as AChE1 or AChE2. The majority of the peptides found in* As*AChE-A suggest that they belong to the AChE-2 while the peptides of* As*AChE-B fit better under AChE-1 (Supporting Information -B, Fig.  S1 and Fig.  S2).

### 3.3. Biochemical Characterization of the Isolated AChEs

**Figure 2:**
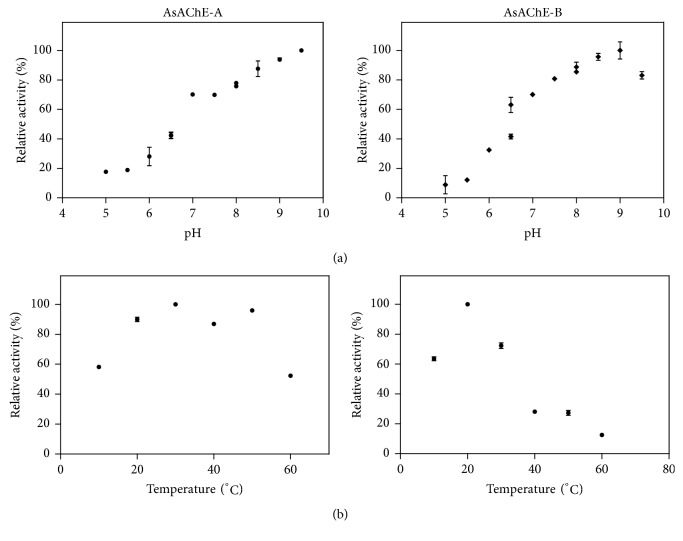
Optimum pH (a) and temperature (b) activities of AsAChE-A and AsAChE-B using ATCh as substrate and the Ellman assay [32].

The influence of the pH and temperature in the activity of free enzymes was determined using ATCh as substrate. With this substrate, AChEs usually have optimum pH around 7.0–8.0. For example, it has been reported that AChEs in* Liposcelis entomophila* has pH around 7.0 [43] while in* H. bacteriophora* the pH values were around 8.5 [41]. Meanwhile, for* As*AChEs, the pH values were above the usual. Optimum pH for* A*sAChE-A and* A*sAChE-B were 9.5 and 9.0, respectively (Figure 2(a)). The optimum temperature for* As*AChE-B was 20°C (Figure 2(b)), as expected for AChEs from insects, but for* As*AChE-A two temperatures (30 and 50°C) gave the maxima of activity (Figure 2(b)). Optimum temperature for AChEs is found in a broad range, varying from 35 to 45°C [41, 43, 44].

The kinetic parameters of the two free enzymes were then determined varying the concentration of ATCh under optimal conditions of pH and temperature. At the analyzed concentration range, typical Michaelian kinetics was observed.

At these conditions, their catalytic efficiency (V_max_/K_M_) was calculated as described by Kim et al. [12]. AChE-A exhibited a V_max_ 3-fold higher than AChE-B and also a lower K_M_ value, which resulted in higher catalytic efficiency. Michaelian kinetic was also observed when ACh was used as substrate and the activities measured by LC-MS; however under these experimental conditions, AsAChE-B emerged as the one with the highest substrate affinity (Table 3).

### 3.4. Preparing AsAChE-ICERs

AsAChE-A and AsAChE-B were efficiently immobilized onto capillary following the same experimental conditions previously published for preparing* eel*AChE-ICERs [39]. The versatility of Vanzolini's activity assay is that the hydrolysis of ACh is monitored by on-flow analysis [39]. Herein, the procedure was adapted, and after method qualification (see Supplementary Section (available here)) the activities of the produced AsAChE-ICERs were measured by LC-MS/MS.

The two produced ICERs showed activity with reproducibility of the assay (n = 2). The initial activity assays using an 85.5 *μ*M solution of ACh provided the following results:* As*AChE-A-ICER produced 14.1 ± 1.1 *μ*M of Ch while* As*AChE-B-ICER produced 64.2 ± 18.1 *μ*M. These results showed, as expected, that the immobilized enzymes retained its activity toward its natural substrate, ACh.

### 3.5. Kinetics Studies of the AsAChE-ICERs and Use of Tacrine as Reference Inhibitor

For the tested concentration range, the curves were best fitted to a Michaelis-Menten hyperbolic function for both AsAChE-ICERs, and, as obtained with the assay in solution, for ACh as substrate, AsAChE-B-ICER (K_M_ = 133.2 ± 24.7) has a higher affinity to the natural substrate ACh than the AsAChE-A-ICER (K_M_ = 188.9 ± 40.0). The kinetic parameters of free and immobilized enzymes should not be directly compared [45], especially in cases where hydrolysis occurs on flow and that the contact time between the enzyme and the substrate is shorter. Thus, *K*_M_ for the AsAChE-ICERs was larger when compared with the assays for the enzymes in solution but followed the same pattern when ACh was used as substrate.

Our results have shown the importance of using the natural substrate. The Ellman method [32], using ATCh as the substrate, is still the most widely used assay either for activity or for prospecting AChEIs [46–50].

For the inhibition screening assay tacrine was selected as reference inhibitor. The reason for selecting this AChEI was based on the well documented results obtained by on-flow assays using immobilized AChEs (human or* E. electricus),* for either ACh [39] or ATCh [44] as substrates, with inhibition in the order of 100%. Herein, tacrine inhibited only 20.0 and 16.0% of* As*AChE-A-ICER and* As*AChE-B-ICER, respectively. Inferring that despite the established use of AChE from* E. electricus* as a model for searching insect AChEIs [51], it is not a completely adequate approach.

## 4. The Cholinergic Function of Isolated AsAChEs

The assays described in the literature to determine the activity of AChEs in the insects generally use ATCh as substrate to infer which main enzyme (AChE1 or AChE2) is involved in the hydrolysis in the synapses [12, 14, 52]. Due to its higher catalytic activity and affinity for ATCh, AChE1 was inferred as the main enzyme involved in the hydrolysis of ACh in the pest insect* Cnaphalocrocis medinalis* [52]. Meanwhile, for* Blattella germanica* (cockroach species), AChE2 was appointed as the main enzyme involved in synapses as it has a greater catalytic efficiency and affinity toward ATCh than AChE1 [12].

Taking this approach into account, the kinetic data obtained using ATCh as substrate suggests that AsAChE-A is the main cholinergic enzyme in* Atta sexdens* which is in agreement with the work carried out by Kim and Lee [17], which shows that all the insects belonging to the order Hymenoptera presented AChE2 as the main enzyme involved in the synapse. Furthermore, our phylogenetic analysis classified AsAChE-A as belonging to the AChE2 class (Supporting Information B).

It is important to stress, however, that the functions attributed to each of the AChEs are not completely clear and that different physiological functions have been assumed to either AChE1 or AChE2 [20].

Studies of the biological functions using RNA interference (RNAi) and gel electrophoresis followed by the enzyme activity test with ATCh in* Tribolium castaneum* (beetle) have suggested that AChE1 is the cholinergic enzyme while AChE2 has been shown to be related to noncholinergic functions, such as embryonic development, growth, and reproduction [19]. The same was observed for grasshoppers [53]. A study carried out in* Helicoverpa armigera* (species) demonstrated that gene silencing resulted in mortality, developmental inhibition, decreased fecundity, and poor formation [18]. In this context, future work using* A. sexdens* AChE RNA interference techniques may elucidate the cholinergic and/or noncholinergic functions of AChE1 and AChE2 in ants. These experiments are necessary, especially considering the apparent kinetic constant obtained for AsAChEs using ACh as substrate.

## Figures and Tables

**Table 1:** Recovery of activity fractions of free enzyme after purification of two AChE from* A. sexdens *using ATCh as substrate.

Procedure	Volume	Total protein	Total activity	Specific activity	Recovery (%)	Purification fold
	(mL)	(mg)	(U*∗*)	(U/mg protein)		
Crude extract	10.0	1915.2	134	0.1	100	1
Ammonium sulfate precipitation	2.5	888.9	241	0.3	180	4
HiTrap DEAE-FF AsAChE-A	2.0	279.7	66	0.2	49	3
HiTrap DEAE-FF AsAChE-B	3.0	87.8	22	0.2	16	4

1U: the amount of enzyme that catalyzes the hydrolysis of 1 *µ*M of ATCh per minute.

**Table 2:** Peptides identified in AsAChE-A and AsAChE-B by mass spectrometry.

Sample	Accession number	Identified protein (organism)	Peptide coverage sequences
AsAChE-A	A0A151I5M4 (KYM90380.1)	AChE (*Atta colombica*)	(1) FAYTGMPTVTETEWPSYTR
			(2) GILQSGTLNAPWSYMTGEKANEVAR
			(3) GILQSGTLNAPWSYmTGEKANEVAR
	A0A158NQX6	Carboxylic ester hydrolase (*Atta cephalotes*)	(4) YSDFLGDEFFVR
			(5) HYFGNEEIAEKTLK
			(6) HYFGNEEIAEK
			(7) SSNPVFPEHPK
	A0A158NS84	Carboxylic ester hydrolase (*Atta cephalotes*)	(8) DQFISAVSELNPYVNQIGR
			(9) SVDAWFGIPYAQKPVGPLR

AsAChE-B	EGI67049.1	AChE (*Acromyrmex echinatior*)	(10) DQFISAVSELNPYVNQIGR
			(11) IVGDYQFTcNVNEFAGR
			(12) YADTGHTVYMYYYK
			(13) HLFNQAIMQSGSATAPWAIISRDESIVR
			(14) GYTHEEIQLSKR
			(15) LAEAVGcPHDR
	EGI59491.1	AChE (*Acromyrmex echinatior*)	(16) FAYTGMPTVTETEWPSYTR
			(17) FAYTGmPTVTETEWPSYTR
			(18) SLEYTDNERDLSLR
	EGI59490.1	AChE (*Acromyrmex echinatior*)	(19) TVLDREVHVFYGVPFAKPPVGPLR
			(20) GILQSGTLNAPWSYMTGEK
	A0A151I5M4 (KYM90380.1)	AChE (*Atta colombica*)	(21) GILQSGTLNAPWSYMTGEKANEVAR
			(22) GILQSGTLNAPWSYmTGEKANEVAR
			(23) TTAcAFWNEFLPR
			(24) YFIWNAEKK

**Table 3:** Kinetic parameters of free enzymes toward ATCh and ACh as substrates.

Substrate	Enzyme	Km	V_max_	Vmax/Km
		(*µ*M)	(*µ*M.min^−1^.mL^−1^)	
ATCh	AsAChE-A	39.1 ± 2.3	23.0 ± 0.4	0.6
	AsAChE-B	52.1 ± 4.7	7.3 ± 0.3	0.1

ACh	AsAChE-A	40.7±12.5	2.3±0.2	0.06
	AsAChE-B	36.0±11.8	1.7±02	0.05

## Data Availability

All the data obtained in the study are shown, discussed, and available in the article.

## Supplementary Materials

Supplementary Materials
* Supporting Information A*.* Analytical Method Validation*. The linearity of the method was evaluated with a standard solution of Ch at 32 mM in water and the working solutions for calibration curves and quality controls (QC) were prepared from that. The stock solutions were prepared at a concentration of 100 *μ*M for the low curve and 25600 *μ*M for the high curve. To prepare the calibration standards and quality control samples, aliquots of 10 *μ*L in the appropriate standard working solutions were added to 90 *μ*L of water. The solutions were homogenised and aliquots of 90 *μ*L were transferred to autosampler vials. A volume of 10 *μ*L from the samples was injected to the Acquity UPLC® BEH HILIC 1.7 *μ*m (2.1 X 100 mm) in LC/TQ-MS system. The calibration standards were prepared in duplicate and the calibration curve was constructed by logarithmic nonlinear regression, plotting the peak area as a function of a given concentration of Ch. The analysis of QC samples allowed the determination of intra- and interbatch with precision and accuracy. Five samples of each concentration were prepared in water. The precision of the method was expressed by the coefficient of variation (CV %) of the replicates. The accuracy was calculated for the concentrations examined by the back calculation and expressed as the percentage of deviation between the concentrations found and the nominal concentrations.* 3.4.1. Qualification Study*. The calibration curves were logarithmic in the concentration ranges studied, with mean correlation coefficients (R^2^) of 0.99 or higher for n =3 (low curve: y = −4,56743 × 10^5^ + 1,32678 × 10^5^ × ln[x + 32,553] and high curve: y = −2,58972 × 10^6^ + 4,64136 × 10^5^ × ln[x + 77,971]). The CV % for the replicates was below 15% and the accuracy showed a deviation below 15% of the nominal value (except for the limit of detection – first point in the curves), showing that no carry over occurred between injections. Accuracy values between 85-112% for low curve and 84-111% for high curve, in the range of accepted criteria. The intra- and interlot precision and accuracy of the method were determined by analyzing five replicates of the three quality controls (QCs), which led to precision values with RSD between 1.1 and 13.7% for the low curve and 1.2 and 2.0% for the high curve. The accuracy of QCs varied between 86.1 and 95.4% for the high curve and 89.3 and 100% for the low curve, indicating that both are in accordance with the criteria for method validation adopted, especially considering that QCs were prepared as replicates (n = 5).* Supporting Information B*. Fig. S1. Phylogenetic tree constructed with AChE1 and AChE2 sequences and the peptides identified by LC-MS/MS for AsChE-A. Fig. S2. Phylogenetic tree constructed with AChE1 and AChE2 sequences and the peptides identified by LC-MS / MS for AsChE-B.
