# Supplementary material for: Acetylcholinesterases from Leaf-Cutting ant Atta sexdens: Purification, Characterization, and Capillary Reactors for On-Flow Assays
Source: Enzyme Res. 2019 Jul 1;2019:6139863. doi: 10.1155/2019/6139863 (PMC6633970; doi:10.1155/2019/6139863)
Supplement: Supplementary Materials — Supporting Information A. Analytical Method Validation. The linearity of the method was evaluated with a standard solution of Ch at 32 mM in water and the working solutions for calibration curves and quality controls (QC) were prepared from that. The stock solutions were prepared at a concentration of 100 μM for the low curve and 25600 μM for the high curve. To prepare the calibration standards and quality control samples, aliquots of 10 μL in the appropriate standard working solutions were added to 90 μL of water. The solutions were homogenised and aliquots of 90 μL were transferred to autosampler vials. A volume of 10 μL from the samples was injected to the Acquity UPLC® BEH HILIC 1.7 μm (2.1 X 100 mm) in LC/TQ-MS system. The calibration standards were prepared in duplicate and the calibration curve was constructed by logarithmic nonlinear regression, plotting the peak area as a function of a given concentration of Ch. The analysis of QC samples allowed the determination of intra- and interbatch with precision and accuracy. Five samples of each concentration were prepared in water. The precision of the method was expressed by the coefficient of variation (CV %) of the replicates. The accuracy was calculated for the concentrations examined by the back calculation and expressed as the percentage of deviation between the concentrations found and the nominal concentrations. 3.4.1. Qualification Study. The calibration curves were logarithmic in the concentration ranges studied, with mean correlation coefficients (R2) of 0.99 or higher for n =3 (low curve: y = −4,56743 × 105 + 1,32678 × 105 × ln[x + 32,553] and high curve: y = −2,58972 × 106 + 4,64136 × 105 × ln[x + 77,971]). The CV % for the replicates was below 15% and the accuracy showed a deviation below 15% of the nominal value (except for the limit of detection – first point in the curves), showing that no carry over occurred between injections. Accuracy values between 85-112% for low curve and 84-111% for high cu [file 6139863.f1.pdf]

Supporting Information A

Analytical Method Validation

The linearity of the method was evaluated with a standard solution of Ch at 32 mM in water, the working solutions for calibration curves and quality controls (QC) were prepared from that. The stock solutions were prepared at a concentration of 100 µM for the low curve and 25600 µM for the high curve. To prepare the calibration standards and quality control samples, aliquots of 10 µL in the appropriate standard working solutions were added to 90 µL of water. The solutions were homogenised and aliquots of 90 µL were transferred to autosampler vials. A volume of 10 µL from the samples was injected to the Acquity UPLC® BEH HILIC 1.7 µm (2.1 X 100 mm) in LC/ TQ-MS system. The calibration standards were prepared in duplicates and the calibration curve was constructed by logarithmic nonlinear regression, plotting the peak area as a function of a given concentration of Ch. The analysis of QC samples allowed the determination of intra- and inter-batch with precision and accuracy. Five samples of each concentration were prepared in water. The precision of the method was expressed by the coefficient of variation (CV %) of the replicates. The accuracy was calculated for the concentrations examined by the back calculation and expressed as the percentage of deviation between the concentrations found and the nominal concentrations.

3.4.1. Qualification Study

The calibration curves were logarithmic in the concentration ranges studied, with mean correlation coefficients (R^2) of 0.99 or higher for n =3 (low curve: $y = -4{,}56743 \times 10^5 + 1{,}32678 \times 10^5 \times \ln[x + 32{,}553]$ and high curve: $y = -2{,}58972 \times 10^6 + 4{,}64136 \times 10^5 \times \ln[x + 77{,}971]$). The CV % for the replicates was below 15 % and the accuracy showed a deviation below 15% of the nominal value (except for the limit of detection – first point in the curves), showing that no carry over occurred between injections. Accuracy values between 85-112% for low curve and 84-111% for high curve, in the range of accepted criteria. The intra- and inter-lot precision and accuracy of the method was determined by analyzing five replicates of the three quality controls (QCs), which led to precision values with RSD between 1.1 and 13.7 % for the low curve and 1.2 and 2.0 % for the high curve. The accuracy of QCs varied between 86.1 and 95.4 % for the high curve and 89.3 and 100 % for the low curve, indicating that both are in accordance with the criteria for method validation adopted, especially considering that QCs were prepared as replicates (n = 5).

Supporting Information B

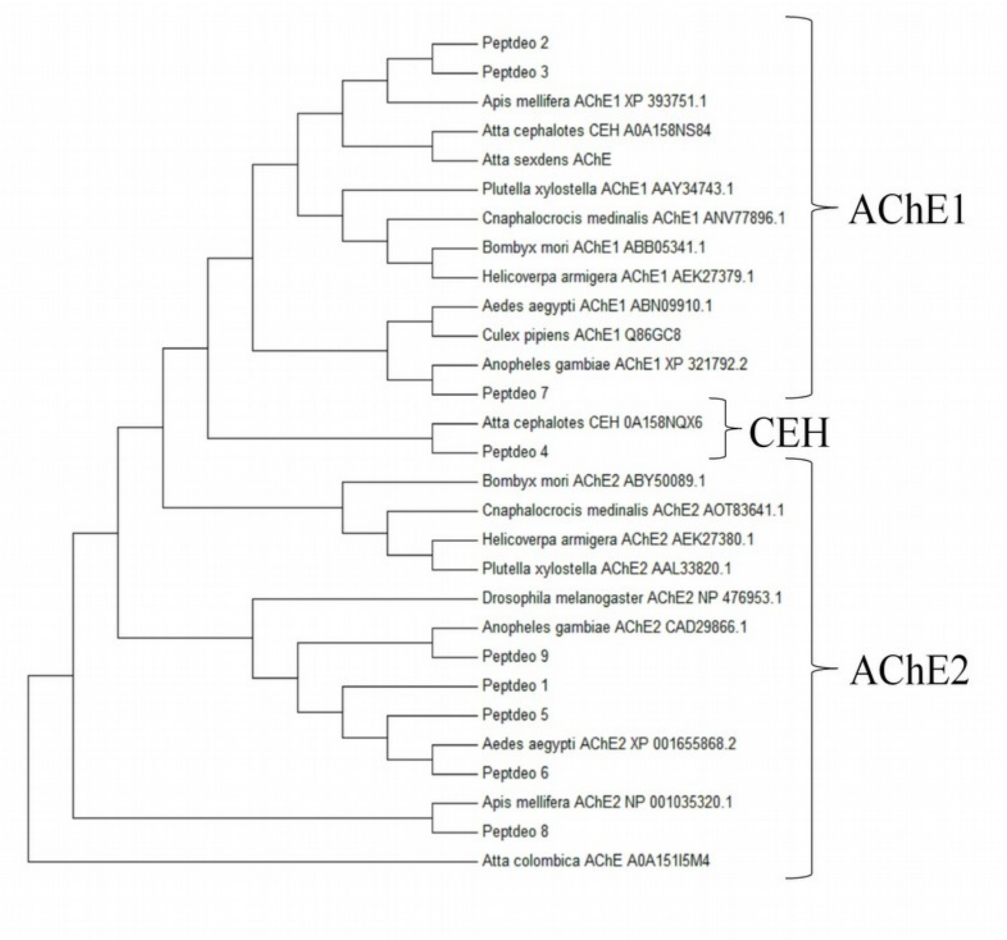

Fig. S1. Phylogenetic tree constructed with AChE1 and AChE2 sequences and the peptides identified by LC-MS / MS for AsChE-A.

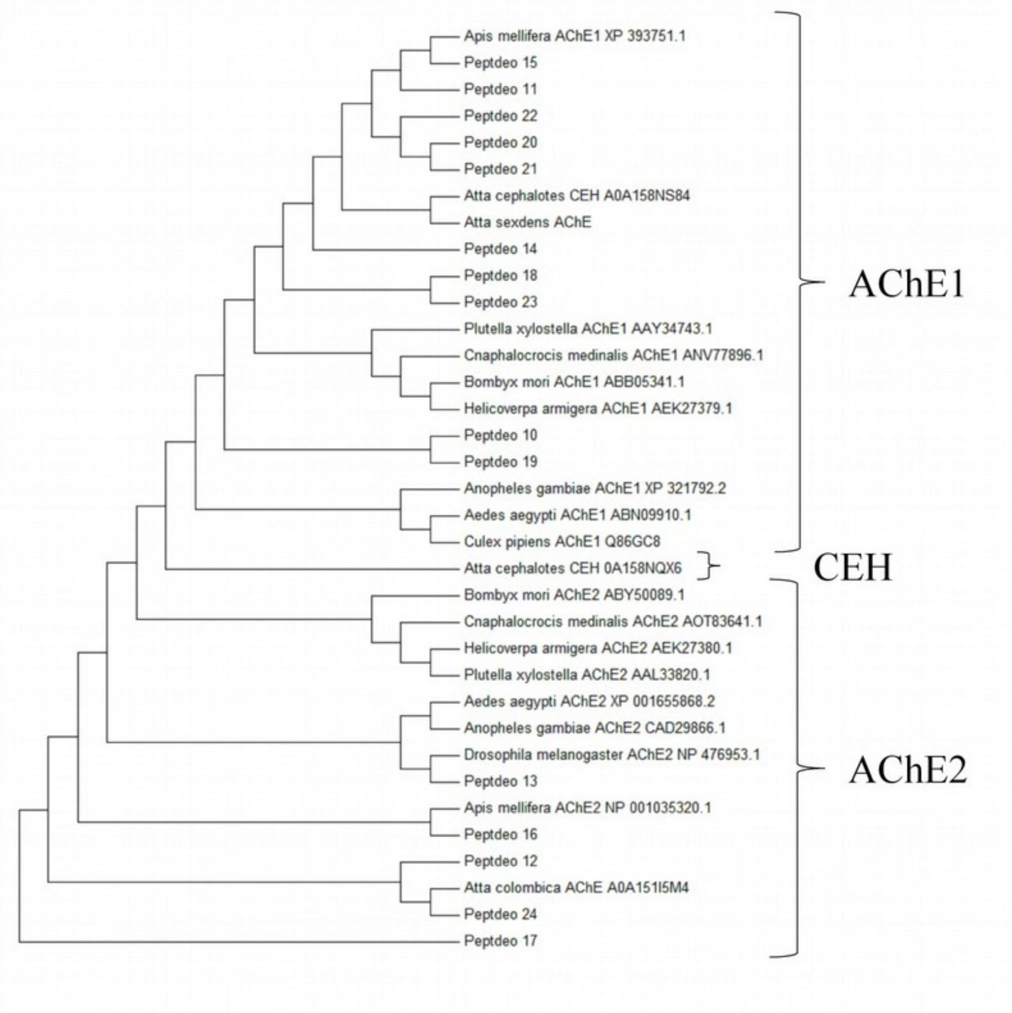

Fig. S2. Phylogenetic tree constructed with AChE1 and AChE2 sequences and the peptides identified by LC-MS / MS for AsChE-B.
